# The role of lysosomal depalmitoylation in Alzheimer's Disease in vitro and in vivo models

**DOI:** 10.1002/alz70855_105339

**Published:** 2025-12-24

**Authors:** Skarleth Cardenas Romero, Mei‐Yu Lai, Matthew J Rosene, Natalia López Herrera, Abraham Lopez, Gabriel Pagan Gonzalez, Adeeb Ahmad, James Patton, Christina Muratore, Bruno A. Benitez

**Affiliations:** ^1^ BIDMC/Harvard Medical School, Boston, MA, USA; ^2^ Beth Israel Deaconess Medical Center, Boston, MA, USA; ^3^ Beth Israel Deaconess Medical Center/Harvard Medical School, Boston, MA, USA; ^4^ Brigham and Women's Hospital, Boston, MA, USA

## Abstract

**Background:**

Amyloidogenic proteins are palmitoylated, and changes in palmitoylation may affect Aβ plaques in AD (Alzheimer's Disease). Palmitoylation enriches amyloidogenic proteins in lipid rafts, while deficiency retains them in the endoplasmic reticulum, inhibiting Aβ accumulation. Palmitoyl protein thioesterase‐1 (PPT1), a key depalmitoylating enzyme, is implicated in AD pathology. We recently showed that *PPT1* hemizygosity increases Aβ plaque formation in the 5xFAD mice. However, the role of lysosomal depalmitoylation in Aβ generation/accumulation/degradation in AD *in vitro* and *in vivo* remains unclear. We tested whether enhancing lysosomal depalmitoylation with a PPPT1‐mimetic could reduce Aβ generation/accumulation in iPSCs‐derived neurons and 5xFAD mice.

**Method:**

We used iPSC with a familial APP mutation associated with AD. The iNs were treated with the NtBuHa PPT1 mimetic for 48 hours and then stained for APP and autophagy/lysosomal markers. Aβ‐40 and Aβ‐42 levels in the media were measured by ELISA. For the 5xFAD mice, NtBuHa (4Mm) was administrated in drinking water for 2 months, with resh drug aliquots given weekly while monitoring weight. We will stain Aβ for plaque load quantification and analyze endo/lysosomal and inflammatory markers in coronal sections (40um). We performed bulk RNA‐seq on cortical homogenates from 5xFAD and P5X mice for DEG and generated SOMAscan data (7000 proteins) for proteomics.

**Result:**

Our preliminary data indicate that 2Mm of NtBuHa significantly reduces Aβ‐40 levels in the media of iNs compared to untreated iNs, with a trend toward decreased Aβ‐42 levels. Thus, there is a trend to increase in the Aβ‐42/ Aβ‐40 ratio compared with the control iNs. We also observed lower expression of APP and Rab7 in iNs treated with 2 Mm of NtBuHa compared to control iNs. Multiple injections of AAV9‐PPT1 into the cortex and hippocampus at 3.5 months of age improved the survival of the animals for at least 18 months. We identified differentially expressed genes and proteins in P5X and 5xFAD mice. Gene expression analysis using the Nanostring metabolic panel (780 genes) revealed that the lysosomal degradation pathway was restored in the AAV2/9‐single‐treated mice.

**Conclusion:**

PPT1 delays AD pathology and induces differential changes in gene and protein levels that affect amyloidogenic pathways activating autophagy/lysosomal processes.

